# Patients with shoulder impingement remain satisfied 6 years after arthroscopic subacromial decompression: A prospective study of 46 patients

**DOI:** 10.3109/17453674.2011.623571

**Published:** 2011-11-25

**Authors:** Karl Lunsjö, Marie Bengtsson, Anders Nordqvist, Fikri M Abu-Zidan

**Affiliations:** ^1^Department of Orthopaedics, Helsingborg Hospital; ^2^Physiotherapist, Primary Health Centre Delta Group, Helsingborg; ^3^Department of Orthopaedics, Skåne University Hospital, Malmö, Sweden; ^4^Department of Surgery, Faculty of Medicine and Health Sciences, UAE University, Al Ain, United Arab Emirates

## Abstract

**Background:**

Although arthroscopic subacromial decompression (ASD) is a common procedure for treatment of shoulder impingement, few long term results have been published. In this prospective study, we determined whether the high degree of patient satisfaction at 6 months postoperatively reported by us earlier remained at the 6-year follow-up.

**Patients and methods:**

We originally reported high patient satisfaction 6 months after ASD for shoulder impingement in 50 prospectively studied patients using the Disability of the Arm Shoulder and Hand questionnaire (DASH) and the Visual Analog Scale (VAS). Patients with associated shoulder disorders were excluded. The surgeons were experienced shoulder arthroscopists. 6 years after surgery, the DASH questionnaire and the VAS were sent to these 50 patients. 2 patients had other medical problems of the upper extremity that affected the DASH and VAS scores, 1 patient was lost to follow-up, and another refused to participate. Thus, 46 patients with a mean age of 55 (33–78) years were included in this 6-year evaluation.

**Results:**

The considerable improvement in both the DASH score and the VAS that was observed 6 months after surgery persisted or had even improved 6 years after surgery.

**Interpretation:**

Properly selected patients with shoulder impingement treated with ASD remain satisfied 6 years after surgery.

Patients with shoulder impingement may have associated conditions such as painful osteoarthrosis of the acromioclavicular joint, degenerative biceps tendon, or rotator cuff tear. There have been few studies describing the long-term results of arthroscopic subacromial decompression (ASD) for patients with pure shoulder impingement, especially from the standpoint of patient satisfaction. The Constant-Murley score (1987) is commonly used; it provides objective information including strength and range of motion. The DASH score reflects the patient's own experience of disability and is used to study rotator cuff disorders (Norlin and Adolfsson 2008, Björnsson et al. 2010). Pain is the main complaint in shoulder impingement patients. Thus, it is important to compare preoperative and postoperative pain to evaluate the surgical results. The VAS is a validated and widely accepted tool that measures the severity of pain.

We have already reported that ASD in properly selected patients with impingement is an operation that gives high patient satisfaction 6 months after surgery when using DASH and VAS as evaluation tools (Bengtsson et al. 2006). We have now evaluated the patients 6 years after surgery.

## Patients and methods

### Original study

In our study from 2006 involving 50 patients, we evaluated patient satisfaction 6 months postoperatively (Bengtsson et al. 2006). We included prospectively collected patients with subacromial pain for more than 6 months without improvement by nonoperative treatment such as subacromial glucocorticosteroid injection and a course of physiotherapy. Radiographs of the humeroscapular and acromioclavicular joints were obtained in all patients. Exclusion criteria were full-thickness rotator cuff tear, instability, clinically verified acromioclavicular joint osteoarthritis, calcifying tendonitis, biceps tendinitis, neurological symptoms, cancer, or not being able to read and understand Swedish. As part of the preoperative evaluation, all patients received an injection of local anesthesia into the subacromial bursa, which should result in pain relief. The operating surgeons were experienced shoulder arthroscopists. Laxity tests were performed when the patients were sedated and before the arthroscope was inserted, to exclude unstable shoulders with secondary impingement. The initial part of the arthroscopy was diagnostic. 5 patients who were included preoperatively were excluded at surgery because of instability in the glenohumeral joint, repair of the rotator cuff, or resection of the lateral end of clavicle. All patients received the same physiotherapeutic treatment protocol postoperatively.

The patients received an envelope with 2 self-assessment instruments (the Swedish version of the DASH questionnaire and the VAS) one week before and 6 months after surgery. The co-author administering the questionnaires (MB) had never met the patients. 50 patients fulfilled the inclusion criteria and completed the questionnaire.

### The 6-year follow-up

6 years after surgery, the DASH questionnaire and the VAS were sent to these 50 patients. The person who administered the questionnaires was not involved in the treatment of the patients. 2 patients had other medical problems of the upper extremity that affected the DASH and VAS scores, 1 patient was lost to follow-up, and another refused to participate. Thus, 46 patients (28 men) were included in the 6-year evaluation. The mean age was 55 (33–78) years.

### Statistics

We used non-parametric statistical methods because of the small number of samples. Friedman test was used to compare DASH and VAS scores before surgery, 6 months after surgery, and 6 years after surgery. If there was any significant difference then the Wilcoxon signed-rank test was used to compare the results between 2 dependent groups. Probabilities of less than 0.05 were considered significant.

## Results

**Figure 1.**
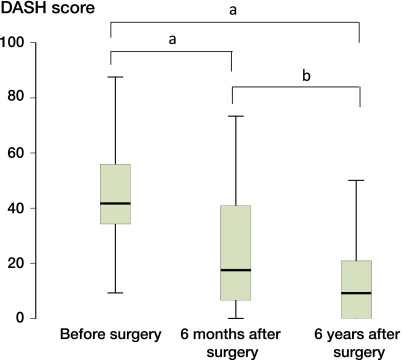
Box plots of DASH score before surgery, 6 months after surgery, and 6 years after surgery. **^a^** p < 0.001 and **^b^** p = 0.02.

**Figure 2.**
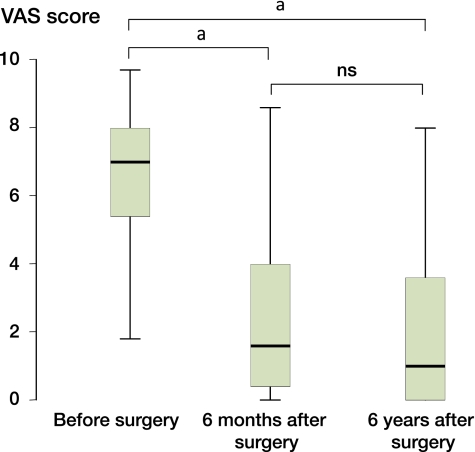
Box plots of VAS score before surgery, 6 months after surgery, and 6 years after surgery. **^a^** p < 0.001.

The DASH and VAS scores improved over the 6 years (p < 0.001) (Figures 1 and 2). The median DASH score improved by 24 points after surgery (median 42 (9–88) before surgery and 18 (0–73) at 6 months postoperatively (p < 0.001). Furthermore, the median DASH score improved by another 9 points at 6 years after surgery (median 9 (0–73)) compared to 6 months after surgery (p = 0.02) (Figure 1).

A mean improvement in DASH score of 19 points has been suggested to be “much better” (Gummesson et al. 2003). A 10-point difference in DASH score may be considered to be the minimal change that is of any clinical importance. This improvement was seen in 37 of the 46 patients 6 years after surgery (relative to the preoperative score). 9 patients did not attain the 10-point drop in DASH score after 6 years. Of these 9 patients, 4 had initially improved at 6 months after surgery but then the patients got worse. 5 patients who did not initially improve by 10 points did so within the 6-year postoperative period.

VAS score had improved at 6 months after surgery (median 1 (0–9)) compared to preoperatively (median 7 (0–10); p < 0.001) (Figure 2). The median VAS score was similar at the 6-year follow-up (median 1 (0–10)) to that at the 6-month follow-up (Figure 2).

## Discussion

We found that most properly selected patients with impingement syndrome remained satisfied 6 years after ASD. In a recent study, Björnsson et al. (2010) followed the rotator cuff status in a series of patients who had had an intact rotator cuff at surgery. They found a low prevalence of cuff tears 15 years postoperatively and concluded that ASD may protect the rotator cuff by reducing the extrinsic factors such as mechanical wear and bursal inflammation. We found further improvement in the DASH score 6 years after surgery compared with 6 months after surgery. As the patients were 6 years older at follow-up, some progression of the degenerative process of the rotator cuff might have been expected (Panni et al. 1996). In a prospective study of 45 patients, Dom et al. (2003) measured Constant score 6 months and 5 years after surgery and found an improvement over time similar to that seen in the present study.

4 of our patients lost their initial improvement in DASH score. We did not determine whether these patients had any partial rotator cuff tear caused by reduced vascularity and degeneration, as reported by Ozaki et al. (1988). An ASD might not prevent this development, especially in patients with the tear on the joint side of the cuff. This may explain why the good results in some of our patients were not maintained over time. Another possible explanation for the loss of improvement seen initially is that the subacromial pain can be attributable to an inflamed bursa that may recur after resection (Santavirta et al. 1992). Henkus et al. (2009) found that patients who had bursectomy alone had results similar to those in patients who had both bursectomy and acromioplasty. These authors concluded that primary subacromial impingement syndrome is largely an intrinsic degenerative condition rather than an extrinsic mechanical disorder.

Pain is the main complaint in shoulder impingement patients, and gives reduced quality of life. The VAS score in our patients was very low at 6 months after surgery and further improvement would not have been expected. Nevertheless, pain levels did not increase. Ketola et al. (2009) measured VAS preoperatively and at 2 years postoperatively in 68 patients treated with ASD, and VAS dropped from 6 to 2. Hoe-Hansen et al. (1999) reviewed 39 patients 6 years after ASD, 13 of whom had intact rotator cuffs at surgery. The mean VAS score was 2, which is slightly higher than the mean score in our patients at 6 years. However, their patient selection was not as strict as ours.

We believe that our result was not affected by the 4 patients who were excluded, because the overall result was not impaired. We used strict patient selection criteria, which plays a major role in determining the surgical outcome. Patel et al. (1999) found a strong correlation between the experience of the surgeon and the outcome. Our surgeons had already passed the learning curve period.

In summary, we have shown that arthroscopic subacromial decompression for impingement, performed by experienced shoulder surgeons after appropriate patient selection, gives high patient satisfaction that is maintained at least up to 6 years after surgery.
